# Identification of the alpha-enolase P46 in the extracellular membrane vesicles of *Bacteroides fragilis*

**DOI:** 10.1590/0074-02760170340

**Published:** 2018-03

**Authors:** Thais Gonçalves Ferreira, Camilla Nunes dos Reis Trindade, Petra Bell, André Teixeira-Ferreira, Jonas E Perales, Rossiane C Vommaro, Regina Maria Cavalcanti Pilotto Domingues, Eliane de Oliveira Ferreira

**Affiliations:** 1 Universidade Federal do Rio de Janeiro, Instituto de Microbiologia Paulo de Góes, Departamento de Microbiologia Médica, Laboratório de Biologia de Anaeróbios, Rio de Janeiro, RJ, Brasil; 2 Fundação Oswaldo Cruz-Fiocruz, Instituto Oswaldo Cruz, Laboratório de Toxinologia, Rio de Janeiro, RJ, Brasil; 3 Rede Proteômica do Rio de Janeiro, Rio de Janeiro, RJ, Brasil; 4 Universidade Federal do Rio de Janeiro, Instituto de Biofísica Carlos Chagas Filho, Laboratório de Ultraestrutura Celular Hertha Meyer, Rio de Janeiro, RJ, Brasil; 5 Universidade Federal do Rio de Janeiro, Duque de Caxias, RJ, Brasil; 6 University of Leeds, Faculty of Biological Sciences, School of Biology, Leeds, UK

**Keywords:** proteomics, Bacteroides fragilis, extracellular membrane vesicles, α-enolase

## Abstract

**BACKGROUND:**

Members of the *Bacteroides fragilis* group are the most important components of the normal human gut microbiome, but are also major opportunistic pathogens that are responsible for significant mortality, especially in the case of bacteraemia and other severe infections, such as intra-abdominal abscesses. Up to now, several virulence factors have been described that might explain the involvement of *B. fragilis* in these infections. The secretion of extracellular membrane vesicles (EMVs) has been proposed to play a role in pathogenesis and symbiosis in gram-negative bacteria, by releasing soluble proteins and other molecules. In *B. fragilis*, these vesicles are known to have haemagglutination and sialidosis activities, and also contain a capsular polysaccharide (PSA), although their involvement in virulence is still not clear.

**OBJECTIVE:**

The aim of this study was to identify proteins in the EMV of the 638R *B. fragilis* strain by mass spectrometry, and also to assess for the presence of Bfp60, a surface plasminogen (Plg) activator, previously shown in *B. fragilis* to be responsible for the conversion of inactive Plg to active plasmin, which can also bind to laminin-1.

**METHODS:**

*B. fragilis* was cultured in a minimum defined media and EMVs were obtained by differential centrifugation, ultracentrifugation, and filtration. The purified EMVs were observed by both transmission electron microscopy (TEM) and immunoelectron microscopy (IM). To identify EMV constituent proteins, EMVs were separated by 1D SDS-PAGE and proteomic analysis of proteins sized 35 kDa to approximately 65 kDa was performed using mass spectrometry (MALDI-TOF MS).

**FINDINGS:**

TEM micrographs proved the presence of spherical vesicles and IM confirmed the presence of Bfp60 protein on their surface. Mass spectrometry identified 23 proteins with high confidence. One of the proteins from the *B. fragilis* EMVs was identified as an enolase P46 with a possible lyase activity.

**MAIN CONCLUSIONS:**

Although the Bfp60 protein was not detected by proteomics, α-enolase P46 was found to be present in the EMVs of *B. fragilis*. The P46 protein has been previously described to be present in the outer membrane of *B. fragilis* as an iron-regulated protein.

Extracellular membrane vesicles (EMVs) are formed by many microorganisms, spanning both prokaryotes (gram-positive and negative bacteria) and eukaryotes (Chernov et al. 2014). Protein secretion into the extracellular environment is a fundamental process in the bacterial communication. There are several secretion pathways described in gram-negative bacteria that can promote the delivery of toxins and other specific proteins to host cells. Among them, are the secretion type III (TSS3) system, for example, which after contact with the cell surface and assembly, can inject effector proteins into host cells. The release of vesicles is involved in the response to environmental stress, virulence factors, the secretion of components destined for the cell surface, antigens, and in the case of pathogens, for host interaction (Deatherage & Cookson 2012).

These vesicles, ranging from 20 to 250 nm in diameter, contain components of the cell wall and outer membrane (OM), flagellin, lipopolysaccharide, cytosine, and pathogen associated molecular patterns (PAMPS) that can activate significantly inflammatory immune responses (Kuehn 2012). In fact, they have provided an alternative for producing acellular vaccines (Avila-Calderón et al. 2012) and this has proven to be effective in the specific case of serogroup B of *Neisseria meningitidis* (Holst et al. 2009).

The majority of gastrointestinal bacteria belong to the Bacteroidetes and Firmicutes phyla. It is well-known that the resident microbiota help in the development of the immune system, and provide a huge number of catabolic enzymes that help to degrade ingested plant polysaccharides. As a member of the intestinal gut flora (Qin et al. 2012), *Bacteroides fragilis* contributes to T helper cell development in particular. However, as a pathogen, it also causes severe infections (abscesses and peritonitis), and is the most frequently isolated gram-negative anaerobic bacterium found in clinical infections (Patrick & Blakely 2012). Several virulence factors have been described in *B. fragilis* to explain this duality within its host. Among them are proteases (Patrick et al. 1996), enterotoxin (Wu et al. 1998), and lipopolysaccharide (Pumbwe et al. 2006), but their roles in pathogenicity have not been elucidated. A capsular polysaccharide (PSA) in the large capsule polysaccharide complex (CPC), produced by *B. fragilis*, has also been shown to have an immunomodulatory effect and to prevent colitis (Shen et al. 2012). PSA is a large molecule, so it has been proposed by Shen et al. (2012) that PSA is released in EMVs and helps to induce immunomodulatory effects and prevent colitis in an animal model. The mechanism by which PSA is delivered to the immune system is still unclear. PSA is however not the only molecule present in EMVs; Patrick et al. (1996) have described the presence of a hemagglutination and enzymatic activity in EMVs, and Domingues et al. (1997) have described a sialidase activity. In 2014, the first antimicrobial molecule, referred to as BSAP-1 (Bacteroidales Secreted Antimicrobial Protein-1), was identified that promotes interference among Bacteroidales strains. BSAP-1 is released in *B. fragilis* EMVs, and contains a membrane attack complex/perforin (MACPF) domain, demonstrating that secreted molecules can promote competitive interference among human gut bacteria (Chatzidaki-Livanis et al. 2014). More recently, Wilson et al. (2015) have identified several putative secretion mechanisms in the outer membrane (OM) of *B. fragilis*, including a family of autotransporters, multiple potential type I secretion system proteins, and possible type VI secretion system proteins.

Bacteria have evolved several virulence strategies to interact with host factors, such as plasminogen (Sun 2006). The fibrinolytic system comprises, among other proteins, plasminogen (Plg), an abundant component of blood that is the zymogenic form of the serine protease plasmin. More than 40 different proteins have been implicated as Plg receptors in pathogenic and commensal bacteria (Sanderson-Smith et al. 2012). In *B. fragilis*, a Plg cell-surface binding protein p60, referred to as Bfp60, was characterized (Ferreira et al. 2013) and shown to be responsible for the conversion of Plg into plasmin and also for laminin-1 recognition. Therefore, the aim of this study was to identify components of the EMVs in *B. fragilis*, using a proteomics approach, and to assess whether Bfp60 was present in these subcellular structures.

## MATERIALS AND METHODS

*Bacterial culture* - The clinical isolate 638R from the rifamycin-resistant *B. fragilis* strain (Privitera et al. 1979) was used in this study. The strain was routinely grown anaerobically in a chamber containing H_2_ (10%), N_2_ (80%), and CO_2_ (10%), using brain heart infusion broth supplemented (BHIS) with hemin (5 mg/mL) and L-cysteine (0.5 g/L) (Jousiemies-Somier et al. 2002). To isolated EMVs, an inoculum was made in a minimum defined medium (MDM) broth containing per litre, 2 g (NH_4_)_2_SO_4_; 0.5 g sodium citrate; 5 mg vitamin B_12_; 7 g KH_2_PO_4_; 8 g K_2_HPO_4_; 10 mg MnCl_2_.4H_2_O; 20 mg MgCl_2_.2H_2_O; 0.3 mg FeCl_3_.6H_2_O; 30 mg CaCl_2_.2H_2_O; 4 g NaHCO_3_; 0.5 g cysteine HCl; 10 g glucose; 5 mg hemin; and 1 mg resazurin (Patrick et al. 1996).

*Purification of EMVs* - In order to obtain a culture enriched in EMVs (Patrick et al. 1996), bacteria were inoculated in MDM broth (500 mL), and the culture was maintained until the late exponential-phase was reached. The culture was then centrifuged at 7245 × *g* using a Beckman GS-6R centrifuge to pellet the bacterial cells. The supernatant fraction was then passed through a 0.45 μm pore-membrane (Millipore) to remove any residual cells, followed by a 0.22 μm polyvinylidene difluoride (PVDF) filter (Millipore). The filtered supernatant was then centrifuged at 30790 × *g* for 4 h using a Sorvall Ultra Pro 80 ultracentrifuge. The supernatant fraction removed and the pellet re-suspended in 1 mL of phosphate buffered saline (0.01 M), pH 7.4 (PBS). The presence of EMVs and the absence of bacterial cells were confirmed by electron microscopy.

*Transmission electron microscopy (TEM) analysis* - Electron microscopy of the EMVs was performed according to the method of Théry et al. (2006). Briefly, 10 μL of the purified vesicles from *B. fragilis* was allowed to settle onto formvar-carbon coated copper grids (300 mesh) for 25 min. After that, the grids were washed twice with phosphate buffered saline (PBS) and fixed with glutaraldehyde (Type I; Sigma-Aldrich Co.) prepared in 0.1 M sodium cacodylate buffer. The grids were negatively stained with 4% uranyl acetate and 2% methylcellulose (1:9) and observed using a Zeiss 900 TEM.

For immunoelectron microscopy, the grids were fixed with 2% paraformaldehyde in PBS for 30 min in a fume hood at room temperature (RT). Following this, the grids were washed four times with PBS containing 100 mM glycine and blocked with 3% bovine serum albumin (BSA, Sigma-Aldrich Co), for 10 min at RT. Before labelling with the antibodies, a solution of 1% BSA and 0.1% saponin in PBS was used to permeabilise the cells for 20 min at RT. The grids were placed in a humidity chamber and incubated with the primary antibody (polyclonal rabbit anti-Bfp60) in PBS containing 1% BSA and 0.1% saponin for 30 min at RT. Grids were washed six times with PBS containing 0.2% BSA and 0.1% saponin, and incubated with a secondary antibody conjugated to 5 nm gold particles (BB International) in PBS containing 0.2% BSA and 0.1% saponin for 1 h. After washing eight times with PBS, the grids were fixed in 1% glutaraldehyde for 5 min and washed eight times with water. Grids were negatively stained with 4% uranyl acetate and 2% methylcellulose (1:9) at 4°C (Théry et al. 2006) and observed with a Zeiss 900 TEM.

*Polyacrylamide gel electrophoresis and western blotting* - EMV proteins were analysed by sodium dodecyl sulphate-polyacrylamide gel electrophoresis (SDS-PAGE) and western blotting. Prior to electrophoresis, the EMV protein concentration was determined using Qubit (Invitrogen), according to the manufacturer's protocol. SDS-PAGE was conducted using a discontinuous bis-acrylamide gel (4% stacking; 12% separating) in a Tris-glycine running buffer (3 g/L Tris, 72 g/L glycine, and 5 g/L SDS) (Laemmli 1970). Protein samples (10 μg) were mixed with sample buffer (Tris-HCl, pH 6.8, 10% SDS, 10% glycerol, 5% 2-b-mercaptoethanol and 0.05% bromophenol blue; Sigma), boiled (100°C) for 5 min, and applied to the SDS-PAGE gel. Protein standards (Spectra Multicolor Broad Range Protein Ladder; Invitrogen) were used as molecular weight markers. The gel was subsequently stained with colloidal Coomassie Blue G-250. Proteins corresponding to a molecular mass range of 35 kDa to approximately 65 kDa were excised from the gel and processed for proteomic analyses (Section 2.5).

For western blotting (Ferreira et al. 2009), proteins were separated by SDS-PAGE and transferred to a nitro-cellulose membrane overnight at 4°C (30 V; 40 mA). The PVDF membrane containing the blotted proteins was washed three times with PBS (0.01 M *, 150 mM NaCl) and blocked with PBST (0.03% Tween 20) containing 5% skimmed milk overnight at 4°C. After washing three times with PBST (0.3% Tween 20), the membrane was incubated with the primary antibody anti-Bfp60 (1:100 for 2 h at RT. The membrane was washed three times with PBST (0.3% Tween 20) and incubated with the secondary antibody anti-rabbit IgG conjugated with peroxidase (1:5000; Sigma) in PBST (0.3% Tween 20) for 1 h at RT. The membrane was then washed three times with PBS and immunoreactive bands were detected by the addition of 50 mg DAB (3,3'diaminobenzidine) dissolved in 30% H_2_O_2_ in 0.01 M PBS. To stop the reaction, the membrane was washed with distilled water.

*In-gel- digestion: mass spectrometry analysis* - After proteins were separated by one-dimensional (1D) SDS-PAGE, bands were excised from the gel and destained in 50% acetonitrile/25 mM ammonium bicarbonate pH 8.0, until no blue colour remained. The gel bands were dehydrated with two incubations of 5 min each in 100% acetonitrile, and air-dried for 10 min, followed by a reduction in 65 mM DTT (30 min at 56°C) and alkylation in 200 mM iodoacetamide (30 min in the dark). Following this, the bands were hydrated with 100 mM ammonium bicarbonate (10 min) and dehydrated twice with 100% acetonitrile (5 min each time), and dried using a Speed-vac (Thermo Scientific™). Digestion was carried out at 37°C overnight in 40 mM ammonium bicarbonate solution containing trypsin (20 ng/μL; Sequencing grade; PromegaÒ). Peptides were extracted in 5% (v/v) formic acid (TFA), 50% (v/v) acetonitrile (30 μL), with the help of ultrasound (10 min). Samples were concentrated to 5 μL and purified with a ZipTip (C18 Millipore®) for direct spotting onto MALDI plates for MALDI-TOF/TOF analysis (Gharahdaghi et al. 1999).

Protein identification by mass spectrometry MALDI-TOF-TOF analysis was performed using a 5800 Proteomics Analyzer (ABSciex). Briefly, 0.5 μL of the micro-column eluate was mixed with 0.5 μL of alpha-cyano-4-hydroxycinnamic acid matrix (20 mg/mL in 50% ACN/0.1% TFA). Samples were spotted onto the ABI 192-targed MALDI plate by co-crystallisation, and mass spectrometry data were acquired in positive reflectron mode, with a mass range 800-3.500 Da. Typically, analyses were conducted using 2.000 shots of MS and 4.000 shots of MS/MS to the ten most abundant ions. External calibration was performed using a mixture of four peptides: des-Arg1-bradykinin (m/z = 904.47), angiotensin I (m/z = 1296.69), Glu1-fibrin peptide B (m/z = 1570.68), and adrenocorticotropic hormone (18-39) (m/z = 2465.20) (mass standards kit for the 4800 Proteomics Analyzer).

*Bioinformatics* - Peptide sequences (protein identity) were determined by matching protein databases with the acquired fragmentation pattern using the software program MASCOT (Matrix Science), and all MS/MS spectra were searched against the entire NCBInr protein database or a modified NCBInr database created to search “fragilis” recognised proteins, assuming use of trypsin as the digestion enzyme, and allowing up two missed cleavages. MASCOT was searched with a product ion mass tolerance of 0.30 Da and a precursor ion tolerance of 0.60 Da. Only significant hits, as defined through MASCOT probability analysis (p < 0.05), and peptides identified with individual ion scores greater than 30 were considered. Scaffold and the Blast2Go (https://www.blast2go.com/b2ghome) were used for functional annotation and analysis with the proteins identified. PsortB v. 3.0 was used to predict the subcellular localisation (www.psort.org/psortb/) and SignalP to predict the presence and location of signal peptide cleavage. Kalign software in the Clustal 2.1 program was used to align the enolase protein sequences.

## RESULTS

**Fig. 1:**
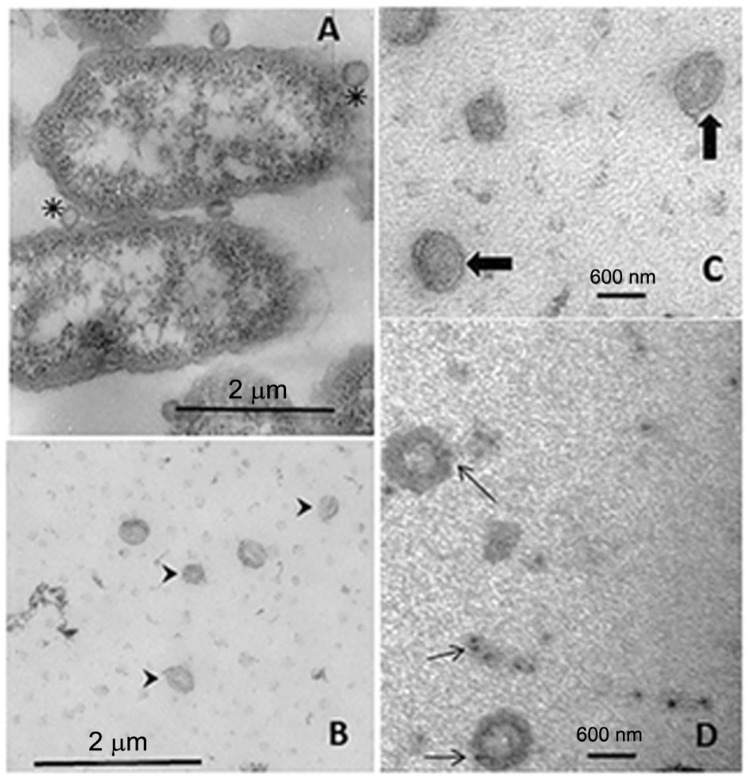
transmission electron microscopy (TEM) of the 638R *Bacteroides fragilis* strain and the extracellular membrane vesicles (EMV) after negative staining with 4% uranyl acetate and 2% methylcellulose. (A) *B. fragilis* with EMVs attached to the cells. The asterisks show the EMVs attached to the surface of the bacteria. Bar = 2 μm. (B-C) TEM of the purified EMVs from the 638R strain. (B) Different sized EMVs (arrow heads) Bar = 2 μm; (C) EMVs magnified with the plasma membrane well-defined (arrows). Magnification = 30,000 ×, Bar = 600 nm; (D) immunogold labelling of a preparation of purified EMVs stained with a rabbit anti-Bfp60 antibody and an anti-rabbit colloidal gold conjugate (5 nm). The arrows indicate staining for the surface protein Bfp60 in the EMVs and cell debris. Magnification = 50,000 ×, Bar = 600 nm.

*Isolation of EMVs and electron microscopy* - EMVs were purified by differential centrifugation from a cell-free culture in a minimum defined medium (MDM), and subsequently pelleted by ultracentrifugation. In order to confirm purification of the EMVs from the *B. fragilis* 638R strain, electron micrographs of EMVs were performed using negative staining. TEM analysis of the purified EMVs revealed them to be attached to the outer surface of *B. fragilis* (Fig. 1A) and consisted of spherical vesicles with a pleomorphic bilayer (Fig. 1B-C). In general, the EMVs released from *B. fragilis* had a circular shape, and were of differing sizes. We then used a polyclonal rabbit anti-Bfp60 antibody (Ferreira et al. 2009) to confirm the presence of the Bfp60 protein on the EMV membrane surface. The immuno-gold labelled TEM micrographs scattered the EMV surface membrane sporadically and also the cell debris (Fig. 1D). The antibody labelling was not as strong as expected, which could indicate lower quantities of Bfp60 in the EMVs or cross-reaction with another surface protein.

**Fig. 2:**
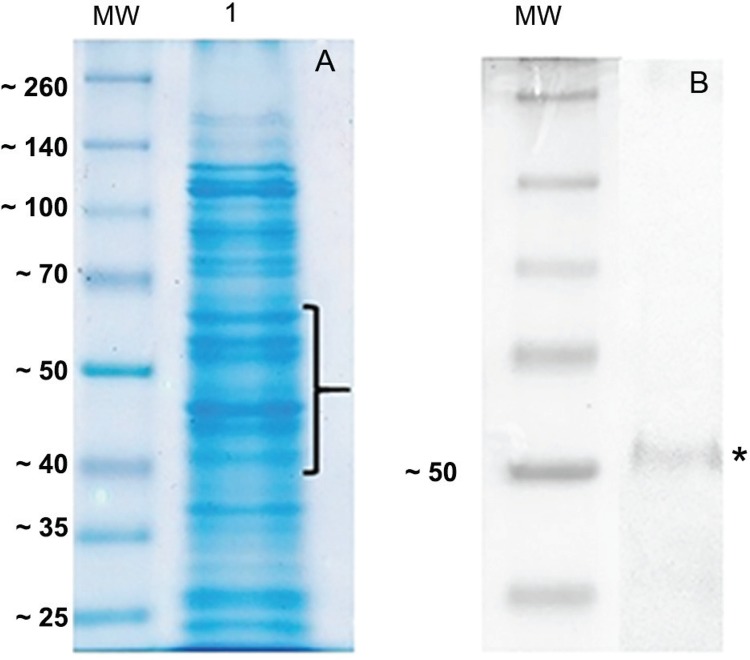
analysis of the protein profile derived from extracellular membrane vesicles (EMVs) isolated from the *Bacteroides fragilis* 638R strain; (A) ten micrograms of purified EMV protein were separated on a 12% SDS-PAGE gel followed by Coomassie colloidal staining (G-250). Lane 1 shows the EMV proteins profile; the brace indicates the selected bands excised from the gel and digested with trypsin. Peptides were enriched using Ziptip C18 columns and analysed by mass spectrometry (Maldi-TOF/TOF). MW indicates the molecular weight standards in kDa. (B) Western-blotting of the EMV protein extract showing the recognition of a protein (asterisk) of approximately 50 kDa by the rabbit anti-Bfp60 antibody. MW indicates the molecular weight standards in kDa.

*SDS-page and western blotting* - The presence of vesicular proteins was analysed by separating 10 μg of total EMV protein by SDS-PAGE and staining with Coomassie blue colloidal stain, which showed the presence of several different proteins (Fig. 2A) consistent with EMV proteins isolated from other gram-negative bacteria (Horstman & Kuehn 2000) as well as *B. fragilis*.

Both western blotting (Fig. 2B) and immunoelectron microscopy (Fig. 1D) confirmed the presence of Bfp60 enolase, however we did not detect the Bfp60 protein in our mass spectrometry analysis. Interestingly, two proteins in the molecular function pie diagram were categorized as having lyase activity. We identified a protein [enolase (Bacteroides); gi number 492241000] that, according to the gene ontology (GO) annotation (Supplementary data, Tables I-II), had enolase activity. A protein blast search with the putative sequence against the NCBI sequence database (http://www.ncbi.nlm.nih.gov/BLAST/) revealed a 100% identity with the P46 α-enolase previously identified in *B. fragilis*. In the *B. fragilis* EMVs this enolase had a cytoplasmic cellular location and molecular weight of approximately 46 kDa (Supplementary data, Tables I-II), the same characteristics found in the inner membrane protein fraction obtained from iron-depleted *B. fragilis* cells.

**Fig. 3:**
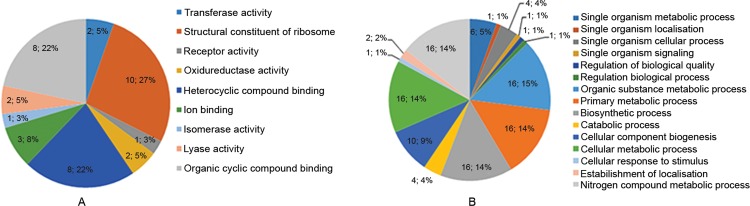
distribution of the *Bacteroides fragilis* extracellular membrane vesicle proteins identified based on gene ontology annotations (GO). (A) Molecular function bar chart graphic and (B) Biological process pie diagram. The Blast2Go software was used to classify the proteins.

*Proteomics analysis* - Duplicate samples of *B. fragilis* 638R EMV proteins were separated using 1D SDS-PAGE and bands ranging from 40 kDa to approximately 65 kDa were excised and digested with trypsin. The extracted peptides were analysed by MALDI TOF/TOF and the data obtained were used to search a database containing the *B. fragilis* genome. The analysis identified 23 proteins, which are listed in Supplementary data, Tables I-II. The distribution of these 23 proteins based on their biological process and molecular function is presented in Fig. 3. Based on the biological process bar chart graphic (Fig. 3B), most of the proteins are involved in binding, act as structural molecules, or possess a catalytic activity. With respect to molecular function (Fig. 3A), the majority of the proteins were structural constituents of the ribosome, which was consistent with the mass spectrometry identification (Supplementary data, Tables I-II).

**Fig. 4:**
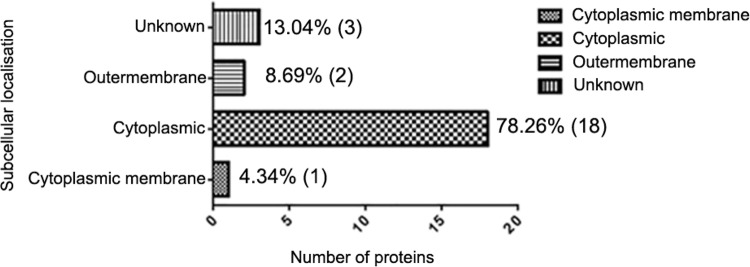
distribution of subcellular locations of proteins identified in *Bacteroides fragilis* extracellular membrane vesicles as determined by PSORTb.

All the EMV proteins identified were subjected to computer analysis using PsortB to identify their predicted protein localisation. The predicted subcellular localisation is shown in Fig. 4. Approximately 78.26% (18) of the proteins were classified as being cytoplasmic with 13.04% (3) being not categorised (unknown). Cytoplasmic membrane (4.04%; 1) and outer membrane (4.04%; 1) proteins were also identified. No periplasmic proteins were identified. Of these 23 proteins, 13.04% were predicted to be secreted (Supplementary data, Tables I-II). Of the proteins containing a signal peptide, three had an uncertain cellular localisation (membrane protein and hypothetic protein) and one was assigned to the outer membrane (putative exported protein).

**Fig. 5:**
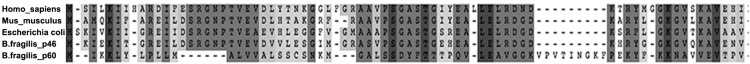
conservation of enolases. Alignment of enolases from human, mouse, *Escherichia coli*, and *Bacteroides fragilis* surface proteins, p46 and p60. The dark shaded residues indicate identity; the light grey residues indicate similarity. Clustal format alignment was made using Kalign.

One of the proteins found in the *B. fragilis* EMVs was identified as an enolase with a possible lyase activity (Supplementary data, Tables I-II). Enolases are highly conserved among organisms, including bacteria and mammalian species. The alignment of enolases from different species, including the P46 and P60 proteins sequences (Fig. 5), suggest that the anti-bfp60 antibody could recognise the P46 α-enolase. In Fig. 5, the dark shaded and light grey residues indicate identity and similarity among the amino acids in the protein sequences, respectively.

## DISCUSSION

Gram-positive and negative bacteria use different types of secretion systems to transport important virulence factors to the cell envelope and the extracellular milieu. One of the mechanisms employed by the bacterial cells to release proteins and mediators is through the shedding and accumulation of membrane vesicles (Théry et al. 2002), which seems to be important process in the intracellular crosstalk between living organisms, including bacteria.

Although several studies examining *B. fragilis* virulence factors have been conducted, there are not many examining EMVs in this species. A preliminary study conducted by Patrick et al. (1996) compared enzymatic activities, including esterase, lipase, alkaline phosphatase, glucosaminidase, and acid phosphatase activities in whole cells and their EMVs in two *B. fragilis* strains, NCTC 9343 and BE3 containing large capsule (LC) and electron-dense layer (EDL) populations. The purified EMVs exhibited both haemagglutination activity and an enzymatic activity suggesting a potential role for the EMV. Domingues et al. (1997) have shown that a sialidase activity was associated with these sub-cellular structures in all the strains analysed, suggesting that these surface components have a function in the commensal stages of *B. fragilis*. More recently (Shen et al. 2012), it was discovered that *B. fragilis* release PSA in EMVs, which induces immunomodulatory effects and prevent colitis in animal models. The authors imply that the EMV-mediated delivery of a commensal molecule prevents disease and provides a mechanism of inter-kingdom communication between the microbiota and mammals. In 2014, Elhenawy et al. (2014) performed a comparative proteomics analysis of the outer membrane vesicles of *B. fragilis* and *B. thetaiotaomicron* where they identified 40 proteins exclusively in *B. fragilis* EMVs. The authors found a high prevalence of glycosidases and proteases, most of the acidic proteins, and active in *in vitro* assays. According to their results the EMVs contained a number of hydrolytic enzymes that were found in both species. Due to the fact that *B. fragilis* and *B. thetaiotaomicron* are members of the human gut microbiota, they can degrade a variety of glycans that are not substrates for human glycosidases. So, the production of short-chain fatty acids generated by the degradation of these glycans can be beneficial to other members of the microbiota. We did not identify any glycosidases in *B. fragilis* EMVs, possibly because, Elhenawy et al. (2014) used a different broth media from ours, enriched with 0.5% glucose or fucose, and employed a different mass spectrometry protocol.

Proteomics approaches have been used previously to determine the components of vesicles in attempts to provide clues to the mechanisms of vesicle production and cargo loading (Lee et al. 2008). In this study, conventional SDS-PAGE coupled to mass spectrometry (MALDI-TOF/TOF) was used to identify the composition of *B. fragilis* EMVs, which extends the list of previously known EMVs protein that are associated with *B. fragilis* pathogenicity.

Although most of the studies (Elmi et al. 2012, Altindis et al. 2014) refer to vesicles being secreted from the bacterial surface, we only observed vesicles attached to the outer membrane of bacterial cells. Spherical vesicles of different sizes were also observed in the micrographs, but no measure of size distribution was made. A membrane lipid bilayer was also observed in the purified EMVs, as has been observed in the EMVs derived from other bacterial species. To check whether the Bfp60 protein was present in the EMVs a polyclonal rabbit anti-Bfp60 enolase antibody was used. The antibody was produced by immunising rabbits with the recombinant Bfp60 enolase protein (Ferreira et al. 2013). Using this antibody, images showed gold labelling on the EMV surface, with gold particles labelling the bacteria EMV, as well as cell debris. Western blotting of total EMV proteins showed a strong recognition of a protein with a mass slightly greater than the 50 kDa molecular weight marker.

Using mass spectrometry we have identified 23 EMV proteins, covering approximately a 35 kDa to 70 kDa molecular weight range. The EMV proteins identified (GI number) and their putative subcellular localisation are listed in Supplementary data, Tables I-II. These proteins were predicted to be involved in transport activity, nutrition, and metabolism, which is consistent with most of the EMVs functions previously identified in bacteria (Elmi et al. 2012). Their predicted subcellular localisations were quite diverse, with proteins predicted to be in the cytoplasmic membrane, the cytoplasmic fraction and the outer membrane. In our analysis, we also obtained evidence for the presence of ribosomal proteins which appeared to be a contamination of the vesicles. We speculate that their presence is due to our protocol of extraction which did not use a density gradient during the purification.

Although numerous studies have described the presence of periplasmic proteins in bacterial EMVs, we did not identify any in this study. This result might be related to the fact that only a limited range of proteins were used in our study, or that there are no periplasmic proteins in *B. fragilis* EMVs. In support of this latter conclusion, a comparative proteomic study of EMVs in *B. fragilis* and *B. thetaiotaomicron* (Elhenawy et al. 2014) did not identify any periplasmic proteins, and instead most of the putative proteins identified by MS/MS in both organisms were putative hydrolases, such as β-glucosidase and β-galactosidase. On the other hand, as previously mentioned, no putative hydrolases were identified in our study. Elhenawy et al. (2014) used a basal medium supplemented with either glucose or fucose. The authors did not give any explanation as to why a supplemented media was used, and there was no reference given to support its specific use, but we assume that the presence of glucose or fucose in the broth might have influenced the production of hydrolases by *B. fragilis* and *B. thetaiotaomicron*.

EMVs are known to contain a full complement of surface antigens, secretory proteins, and toxins (Deatherage & Cookson 2012). The present study tried to identify the presence of an outer membrane protein, Bfp60 (p60) which has been characterized as an enolase, in *B. fragilis*. The proteomics analysis that we performed identified several proteins in the *B. fragilis* EMVs; among these was the P46 enolase. Enolase is an ancient and ubiquitous metalloenzyme, which catalyses the reversible dehydration of 2 phospho D-glycerate into phosphoenolpyruvate during glycolysis. Eubacteria and archaebacteria both contain a single enolase gene and therefore multiple molecular forms (isoforms) are not observed. Overall, enolase sequences are highly conserved, showing a roughly 40% amino acid identity across eukaryotes, eubacteria, and archaebacteria, although a large number of insertions and deletions occur across phyla. When enolases from different species (*Homo sapiens*, *Mus musculus*, *Escherichia coli*, and *B. fragilis*) were aligned, P46 showed a good degree of amino acid similarity with the human, mice, and *E. coli* sequences. In contrast, the Bfp60 amino acid sequence did not show the same similarity, although some conserved regions (homology) could explain why the polyclonal antibody recognised a protein by western blotting. Enolases are dimeric hydrolases encoded by three genes, alpha (α), beta (β), and gamma (γ) (Brown & Doolittle 1997). These enzymes were initially studied only in neuronal, cancerous, and hematopoietic cells (Nakagima et al. 1994). How α-enolase is transported through the cell membrane and sorted to the cell surface without the presence of a signal sequence remains intriguing (Muesch et al. 1990). The presence of α-enolase at the cell surface, where it acts as a receptor and not an enzyme is not surprising because there is an ever-growing list of proteins which are targeted to more than one location in both prokaryotes and eukaryotes with variable biological functions.

In conclusion, we have identified 23 *B. fragilis* EMVs proteins, to our knowledge, the most comprehensive proteomic analysis related to this structure. Although we did not identify the Bfp60 protein, we believe that with further studies, *B. fragilis* virulence factors secreted within these EMVs will help us to comprehend the participation of those EMVs contents during pathogenesis, modulation of the host cell response to *B. fragilis* infection, and the establishment and maintenance of the species in the human microbiota. In addition, we have provided new insights in a biological field that is poorly characterised, namely that of commensal bacteria which are a major component of the human gut microbiome.
