# Supplementary material for: Identification of the alpha-enolase P46 in the extracellular membrane vesicles of Bacteroides fragilis
Source: Mem Inst Oswaldo Cruz. 2018 Mar;113(3):178–84. doi: 10.1590/0074-02760170340 (PMC5804310; doi:10.1590/0074-02760170340)
Supplement: Supplementary file 1 [file 0074-0276-mioc-113-03-0178-Suppl01.pdf]

TABLE I

Bacteroides fragilis extracellular membrane vesicle proteins identified

Number of proteins	GI number	Gene symbol	Protein description	Subcellular location	Size	MW (kDa)	Signal peptide
1	496044866	*susD*	Putative outer membrane receptor protein probably involved in nutrient binding [*Bacteroides fragilis* YCH46]	Cytoplasmic membrane	504 aa	56.7	NO
3	499302510	*susD*	Membrane protein	Unknown	539 aa	60.2	YES
4	492236965	*susD*	MULTISPECIES: hypothetical protein [*Bacteroides*] (Outer membrane protein- membrane bound beta-barrel fold)	Unknown	208 aa	23.2	YES
5	492233537	*cysK*	Cysteine synthase [*Bacteroides*]	Cytoplasmic	325 aa	32.7	NO
6	492233692	*BF0087*	Serine acetyltransferase [*Bacteroides*] (hexapeptide family protein)	Cytoplasmic	299 aa	32.4	NO
7	492247116	*ftnA*	Non-heme Ferritin A [*Bacteroides*]	Cytoplasmic	159 aa	17.9	NO
8	492238335	*gap*	Glyceraldehyde-3-phosphate dehydrogenase [*Bacteroides*]	Cytoplasmic	333 aa	35.5	NO
9	492241000	*eno*	Enolase [*Bacteroides*]; the export of enolase possibly depends on the covalent binding to the substrate; once secreted, it remains attached to the bacterial cell surface.	Cytoplasmic	429 aa	46.2	NO
10	492247603	*pgk*	Phosphoglycerate kinase [*Bacteroides*]	Cytoplasmic	419 aa	44.9	NO
11	492254727	*tpiA*	Triosephosphate isomerase [*Bacteroides*]	Cytoplasmic	251 aa	26.4	NO
12	492268887		Conserved hypothetical exported protein [*Bacteroides fragilis*]	Unknown	297 aa	33.8	NO
13	496044166		Putative exported protein [*Bacteroides*]	Outer-membrane	244 aa	27.8	YES
14	492228638	*rpsC*	30S ribosomal protein S3 [*Bacteroides*] (Binds the lower part of the 30S subunit head. Binds mRNA in the 70S ribosome, positioning it for translation)	Cytoplasmic- small ribosomal subunit	244 aa	27	NO
15	492255195	*rpsD*	30S ribosomal protein S4 [*Bacteroides*]	Cytoplasmic- small ribosomal subunit	201 aa	22.4	NO
16	492228631	*rpsS*	30S ribosomal protein S19 [*Bacteroides*]	Cytoplasmic- small ribosomal subunit	89 aa	9.7	NO
17	488621337	*rpsM*	30S ribosomal protein S13 [*Bacteroides*]	Cytoplasmic- ribosome	126 aa	13.9	NO
18	492255420	*rpsB*	30S ribosomal protein S2 [*Bacteroides*] (“putative laminin-1 binding site”)	Cytoplasmic- small ribosomal subunit	278 aa	30.3	NO
19	492255156	*rpsG*	30S ribosomal protein S7 [*Bacteroides*]	Cytoplasmic- small ribosomal subunit	158 aa	18	NO
20	492228614	*rpsJ*	30S ribosomal protein S10 [*Bacteroides*]	Cytoplasmic- ribosome	101 aa	11.3	NO
21	488621337	*rpsM*	30S ribosomal protein S13 [*Bacteroides*]	Cytoplasmic- ribosome	126 aa	13.9	NO
22	492246710	*rpsP*	30S ribosomal protein S16 [*Bacteroides*]	Cytoplasmic- ribosome	181 aa	19.4	NO
23	492255784	*rpsO*	30S ribosomal protein S15 [*Bacteroides*]	Cytoplasmic- ribosome	89 aa	10.2	NO
24	491925255	*rpsL*	30S ribosomal protein S12 [*Bacteroides*]	Cytoplasmic- small ribosomal subunit	133 aa	14.6	NO

TABLE II

GO Biological process	GO Molecular function	Function
Cysteine biosynthetic process from serine	Cysteine synthase activity; transferase activity	Catalytic activity: O(3)-acetyl-L-serine + H(2)S = L-cysteine + acetate; similarity: belongs to the cysteine synthase/cystathionine beta-synthase family
Sulphur and cysteine metabolism	Serine O-acetyltransferase activity	
Cellular iron ion homeostasis and iron ion transport	Ferric iron binding	Contains 1 ferritin-like di-iron domain
Glucose metabolic process	NAD and NADP binding; oxidoreductase activity, acting on the aldehyde or oxo group of donors, NAD or NADP as acceptor	Belongs to the glyceraldehyde-3-phosphate dehydrogenase family
Phosphopyruvate hydratase activity	Lyase activity	Catalytic activity: 2-phospho-D-glycerate = phosphoenolpyruvate + H(2) O; cofactor: magnesium. Required for catalysis and for stabilizing the dimer; enzyme regulation: the covalent binding to the substrate causes inactivation of the enzyme, and possibly serves as a signal for the export of the protein; function: catalyses the reversible conversion of 2-phosphoglycerate into phosphoenolpyruvate. It is essential for the degradation of carbohydrates via glycolysis; pathway: carbohydrate degradation; glycolysis; pyruvate from D-glyceraldehyde 3-phosphate: step 4/5; similarity: belongs to the enolase family; subcellular location: fractions of enolase are present in both the cytoplasm and on the cell surface. The export of enolase possibly depends on the covalent binding to the substrate; once secreted, it remains attached to the bacterial cell surface.
Glycolysis	ATP binding; phosphoglycerate kinase activity	Catalytic activity: ATP + 3-phospho-D-glycerate = ADP + 3-phospho-D-glyceroyl phosphate; pathway: carbohydrate degradation; glycolysis; pyruvate from D-glyceraldehyde 3-phosphate: step 2; similarity: belongs to the phosphoglycerate kinase family.
Gluconeogenesis; glycolytic process; pentose-phosphate shunt	Triose-phosphate isomerase activity	
Translation	mRNA binding; rRNA binding; structural constituent of ribosome	Binds the lower part of the 30S subunit head. Binds mRNA in the 70S ribosome, positioning it for translation; similarity: belongs to the ribosomal protein S3P family; similarity: contains 1 KH type-2 domain; subunit: part of the 30S ribosomal subunit. Forms a tight complex with proteins S10 and S14.
Translation	rRNA binding; structural constituent of ribosome	One of the primary rRNA binding proteins, it binds directly to 16S rRNA where it nucleates assembly of the body of the 30S subunit; function: with S5 and S12 plays an important role in translational accuracy; similarity: belongs to the ribosomal protein S4P family; similarity: contains 1 S4 RNA-binding domain; subunit: part of the 30S ribosomal subunit. Contacts protein S5. The interaction surface between S4 and S5 is involved in control of translational fidelity.
Translation	rRNA binding; structural constituent of ribosome	Protein S19 forms a complex with S13 that binds strongly to the 16S ribosomal RNA; similarity: belongs to the ribosomal protein S19P family.
Translation	rRNA binding; structural constituent of ribosome; tRNA binding	
Translation	Structural constituent of ribosome	Belongs to the ribosomal protein S2P family
Translation	rRNA binding; structural constituent of ribosome; tRNA binding	One of the primary rRNA binding proteins, it binds directly to 16S rRNA where it nucleates assembly of the head domain of the 30S subunit. Is located at the subunit interface close to the decoding centre, probably blocks exit of the E-site tRNA; similarity: belongs to the ribosomal protein S7P family; subunit: part of the 30S ribosomal subunit. Contacts proteins S9 and S11.

GO Biological process	GO Molecular function	Function
Translation	Structural constituent of ribosome; tRNA binding	Involved in the binding of tRNA to the ribosomes; similarity: belongs to the ribosomal protein S10P family
Translation	Structural constituent of ribosome; tRNA binding	Located at the top of the head of the 30S subunit, it contacts several helices of the 16S rRNA. In the 70S ribosome it contacts the 23S rRNA (bridge B1a) and protein L5 of the 50S subunit (bridge B1b), connecting the 2 subunits; these bridges are implicated in subunit movement. Contacts the tRNAs in the A and P-sites; similarity: belongs to the ribosomal protein S13P family; subunit: part of the 30S ribosomal subunit. Forms a loose heterodimer with protein S19. Forms two bridges to the 50S subunit in the 70S ribosome
Translation	Structural constituent of ribosome	Belongs to the ribosomal protein S16P family.
Translation	rRNA binding; structural constituent of ribosome	Forms an intersubunit bridge (bridge B4) with the 23S rRNA of the 50S subunit in the ribosome; function: one of the primary rRNA binding proteins, it binds directly to 16S rRNA where it helps nucleate assembly of the platform of the 30S subunit by binding and bridging several RNA helices of the 16S rRNA; similarity: belongs to the ribosomal protein S15P family; subunit: part of the 30S ribosomal subunit. Forms a bridge to the 50S subunit in the 70S ribosome, contacting the 23S rRNA.
Translation	rRNA binding; structural constituent of ribosome; tRNA binding	Interacts with and stabilizes bases of the 16S rRNA that are involved in tRNA selection in the A site and with the mRNA backbone. Located at the interface of the 30S and 50S subunits, it traverses the body of the 30S subunit contacting proteins on the other side and probably holding the rRNA structure together. The combined cluster of proteins S8, S12 and S17 appears to hold together the shoulder and platform of the 30S subunit; function: with S4 and S5 plays an important role in translational accuracy; similarity: belongs to the ribosomal protein S12P family; subunit: part of the 30S ribosomal subunit. Contacts proteins S8 and S17. May interact with IF1 in the 30S initiation complex.
